# Evaluation of Antithrombogenicity and Hydrophilicity on Zein-SWCNT Electrospun Fibrous Nanocomposite Scaffolds

**DOI:** 10.1155/2012/345029

**Published:** 2012-02-08

**Authors:** Brahatheeswaran Dhandayuthapani, Saino Hanna Varghese, Ravindran Girija Aswathy, Yasuhiko Yoshida, Toru Maekawa, D. Sakthikumar

**Affiliations:** Bio-Nano Electronics Research Center, Graduate School of Interdisciplinary New Science, Toyo University, 2100 Kujirai, Saitama, Kawagoe 350-8585, Japan

## Abstract

Design of blood compatible surfaces is required to minimize platelet surface interactions and increase the thromboresistance of foreign surfaces when they are used as biomaterials especially for artificial blood prostheses. In this study, single wall carbon nanotubes (SWCNTs) and Zein fibrous nanocomposite scaffolds were fabricated by electrospinning and evaluated its antithrombogenicity and hydrophilicity. The uniform and highly smooth nanofibers of Zein composited with different SWCNTs content (ranging from 0.2 wt% to 1 wt%) were successfully prepared by electrospinning method without the occurrence of bead defects. The resulting fiber diameters were in the range of 100–300 nm without any beads. Composite nanofibers with and without SWCNT were characterized through a variety of methods including scanning electron microscopy, transmission electron microscopy, thermogravimetric analysis, and tensile mechanical testing. The water uptake and retention ability of composite scaffolds decreased whereas thermal stability increased with an addition of SWCNTs. Hemolytic property and platelet adhesion ability of the nanocomposite (Zein-SWCNTs) were explored. These observations suggest that the novel Zein-SWCNTs composite scaffolds may possibly hold great promises as useful antithrombotic material and promising biomaterials for tissue engineering application.

## 1. Introduction

Implantation always involves penetration to the skin and damage of smaller or larger blood vessels through the surgical procedure and the implanted scaffold material will have direct contact with human blood. Scaffolds as medical implants require assessment of hemocompatibility to predict potential procoagulation or immune system activity [1, 2]. Antithrombogenic biomaterial is being extensively studied in order to fabricate artificial organs and biomaterials in contact with blood. A significant goal for the application of antithrombogenic biomaterial is to prevent thrombus formation on the material surface. In general, the factors, which influence the hemocompatibility of a material, include the chemical structure of the surface, the hydrophobicity/hydrophilicity, surface charge, surface roughness and the thrombus formation when the surface is in contact with blood [3]. The design of a suitable biomaterial that offers improved blood biocompatibility is highly desirable for blood-contacting devices.

The fabrication and design of submicron to nanoscale structural architectures, which geometrically or topologically mimic the native state of extra cellular matrix, have received much attention in regenerative medical applications [4]. Electrospinning has recently emerged as a leading technique for the formation of nanofibers because it can produce fibers with diameters ranging from several microns to tens of nanometer depending upon the solution and process parameters [5]. Nanofibrous scaffolds have attracted lot of attention as scaffolds for tissue engineering applications due to their structural similarity to extra cellular matrix, having very large surface area to volume ratio, flexibility in surface functionality, superior mechanical properties, and its high porosity [5, 6]. 

Zein is a protein present in corn in the seeds of maize in large amounts. Due to its biodegradability and biocompatibility [7], researchers have provided proofs that Zein was also used as a biomaterial in various biomedical applications. The proposed uses of Zein in biomedical applications are as carriers for drug delivery [8–10], food packaging [11], and scaffolding materials for cell/tissue culture [7, 12]. However the mechanical strength of the electrospun Zein scaffold was found to be very low. In an effort to improve the tensile properties of zein nanofiber, we have incorporated zein with the SWCNT.

Due to the unique properties of CNTs such as mechanical, electrical, thermal, optical, and structural properties, currently researchers have been being explored its applications in biomedical engineering and medical chemistry [13, 14]. Researchers have focused on utilizing these remarkable characteristics for engineering applications such as polymeric composites, materials for energy storage [15], electronics [16], catalysis [17], and vaccine delivery [18]. A recent report on safety issues and toxic effects of CNTs argued that exposure to pristine CNTs causes minimal cytotoxicity at higher concentrations, while chemically functionalized CNTs did not show any toxicity for drug delivery applications [19]. Furthermore, the cell growth on CNTs-based materials has been shown to change cell behavior, such as migration and viability due to the influence of electric fields [20]. CNTs have been claimed to suffer the demerit of not being biodegradable [21]. However, it has been shown that CNTs can be discharged from the body without any side effect or mortality [22].

In recent years, considerable attention has been focused on preparing nanocomposites wherein CNTs are dispersed in different polymeric matrices [23–25]. The dispersion of nanometer-sized materials in the polymer medium was suggested as an effective method to increase the mechanical and electrical properties of the system [26–28]. One promising candidate as the nanocomposite component is the SWCNTs. These composites may consist of synthetic polymers [29, 30], naturally derived biopolymers [31] or a combination of both [32]. The reinforcement of naturally derived polymers with CNTs is another promising area that is just beginning to be explored. Up to date, the nanoscale fillers and polymer composite reinforced with electrospun nanofibers have been developed mainly for providing some outstanding physical (e.g., optical and electrical) and chemical properties and provides superior structural properties such as high modulus and strength to weight ratios. The rigidity and cylindrical shape of CNTs make their surfaces good supports for protein crystallization [33] which can improve the mechanical properties of the electrospun nanofibers. Supronowicz and Webster et al., have observed that the potential use of composites of synthetic polymers and CNT in neural and orthopedic tissue engineering applications [34, 35]. The inert nature and biocompatible chemical surfaces to cells and tissues, multiwalled carbon nanotubes—polyurethane composite [36] and poly(carbonate urethane) [37] have been demonstrated to have excellent antiadhesion to platelets. 

Inspired by these promising results, we aim to develop a novel nanofibrous blood-compatible scaffold incorporating CNTs for its reduced thrombogenicity effect and with improved mechanical strength. In addition, electrospinning method offers the synthesis polymer/carbon nanotubes (CNTs) nanocomposite fibers without compromising the structural integrity of the individual CNTs [38, 39].

In this study, we have attempted the coelectrospinning method to prepare SWCNTs-Zein nanofiber with different SWCNTs content (ranging from 0.2 wt% to 1 wt%) through a solution-based method, which has the potential of providing much better dispersion of SWCNTs on the polymer matrix yielding a composite with uniform structure. We are reporting here the effect of SWCNTs content on the morphology, mechanical properties thermal stability, and its excellent antiadhesion to platelets of Zein-SWCNT nanocomposite scaffolds. We found that Zein-SWCNT composite containing 22 wt% zein and 0.8 wt% SWCNT is very good candidate for biomaterial applications.

## 2. Materials and Methods

### 2.1. Materials

Zein (Wako pure chemicals Industries LTD., Japan), SWCNT was obtained from Sigma-Aldrich. The solvent used Trifluoro ethanol (TFE) was purchased from Kanto Chemical Co. (Tokyo, Japan) used as such without further purification.

### 2.2. Preparation of Spinning Dope Solutions

The spinning solutions were prepared from single-solvent system. Zein was dissolved in TFE (100%) to form a homogeneous solution and maintained under constant stirring for 24 hrs. The spinning dope was prepared by sonicating 0.2–1 wt% SWCNTs in TFE for 2 hrs. Zein (22 wt%) was added to the SWCNT-TFE mixture and further sonicated for an hour and then stirred for another hour.

### 2.3. Electrospinning Process

To fabricate the ultrafine composite nanofibrous scaffold we have used NANON electrospinning setup (NANON-01A, MECC Co., Ltd., Fukuoka, Japan). For electrospinning, 10 mL of each kind of Zein-SWCNTs solution was loaded into a 10 mL glass syringe and injected through an 18 G stainless-steel blunt-ended needle. The syringe was then placed in a syringe pump at a flow rate of 0.5 mLh^−1^. The electric field 2.0 kVcm^−1^ (expressed in terms of voltage/distance) between the collection plate (cathode) and the needle tip (anode) was applied. The collector covered with aluminum foil placed at 10 cm on which fibers were collected. All electrospinning processes were performed at ambient temperature.

### 2.4. Characterization of Nanocomposite Scaffold

The morphology and diameters of electrospun fibers were determined using scanning electron microscope (JSM-7400F, JEOL, Japan). The sample was sputter coated with a thin platinum layer using an autosputter fine coater (E-1030 Ion sputter, Hitachi, Japan) before imaging. According to the SEM images, the diameter and distributions of ultrafine fibers were measured. Additionally, Zein-SWCNT composite fibers were examined in a TEM (JEM 2100, JEOL, Japan). The samples were prepared by drawing out small fibrils with the aid of tweezers and placed on carbon-coated copper grids. Samples were stained by exposure to osmium tetroxide vapour for one hour. The accelerating voltage used was 120 kV.

### 2.5. Thermal Properties of Electrospun Zein-SWCNTs Nanocomposite

To measure the thermal properties of the electrospun nanocomposite fibers, the thermal stability was analyzed in a thermogravimetric analyzer (TGA) (DTG-60H, Shimadzu, Japan). The runs were performed in the temperature range of 30°C to 1000°C and consisted of a ramp at a steady rate of 10°C min^−1^ with continuous nitrogen flow.

### 2.6. Mechanical Properties of Electrospun Zein-SWCNTs Nanocomposite

The tensile properties of electrospun nanocomposite fibrous scaffolds, approximately 8 × 0.03 × 25 mm^3^ (*L* × *W* × *H*), were characterized using a Universal Testing Machine (Instron 3345, UK) equipped with a 500 N load cell. The ends of the rectangular specimens were mounted vertically on two mechanical gripping units of the tensile tester, leaving a 40 mm gauge length for mechanical loading at an extension rate of 1 mm min^−1^. The reported tensile moduli and maximum tensile strengths represented average results of five tests.

### 2.7. Evaluation of Hydrophilicity

The water uptake was determined gravimetrically. The weights of the completely dried films were determined directly with an analytical balance (*W*_dry_). Strips of Zein-SWCNT-based scaffold (1 cm × 2 cm) were immersed into deionized water at 37°C in an incubator for 24 h. The resultant swollen scaffold was gently blotted with filter paper to remove excess surface water and weighed again (*W*_wet_). The water uptake (WT) of the scaffold is expressed as the percentage of weight obtained using (1):

(1) $$\text{WT\%}=\frac{\;W_{\text{wet}}-W_{\text{dry}}}{W_{\text{dry}}}\times 100.$$

To measure the water retention ability, the wet scaffolds were transferred to centrifuge tubes with filter paper at the bottom, centrifuged at 500 rpm for 3 m and weighed immediately (*W*_wet_). The percentage of water retention (WR) of the scaffolds at equilibrium was calculated using following (2):

(2) $$\text{WR}\%=\frac{W_{\text{wet}(R)}-W_{\text{dry}}}{W_{\text{dry}}}\times 100,$$

where *W*_wet(*R*)_ is the wet weight after a predetermined time, *W*_dry_ is the original weight of the sample.

### 2.8. Hemolysis Analysis

The nanocomposite scaffolds (Zein nanofiber; Zein-0.2 wt% SWCNT; Zein-0.5 wt% SWCNT; Zein-0.8 wt% SWCNT; Zein-1 wt% SWCNT) were cut into small pieces and the samples were equilibrated in normal saline water for 30 min at room temperature. Rabbit blood obtained from Nihon Seibutsu Zairyo Centre, Japan. 200 *μ*L was added to each scaffold after taking dry weight of each scaffold. After a predetermined time period, 4 mL of saline water added to each sample to stop hemolysis and the samples were kept at constant temperature (35°C) for 1 h. Positive and negative controls were produced by adding 200 *μ*L of rabbit's blood to 4 mL of distilled water and saline water respectively. All the test samples were centrifuged. Optical density (OD) of the supernatant was measured at 545 nm using spectrophotometer (V-650 spectrophotometer, Jasco, Japan). The experiments were run in triplicate and were repeated twice. The percent of hemolysis was calculated using (3):

(3) $$\begin{array}{cc} & \text{\%}\;\text{Hemolysis}\;\left(\text{HP}\right)\\ & \;\;=\frac{\text{OD}\;\text{of}\;\text{test}\;\text{sample}-\text{OD}\;\text{of}\;–\text{ve}\;\text{control}}{\text{OD}\;\text{of}\;+\text{ve}\;\text{control}-\text{OD}\;\text{of}\;–\text{ve}\;\text{control}}\times 100. \end{array}$$

### 2.9. Platelet Adhesion Examination

The platelet adhesion study was performed according to International standard 10993-4 [40]. For platelet adhesion studies, nanocomposite scaffolds of 1.5 × 1.5 cm size were used. Experiments were carried out with anticoagulated rabbit blood bought from the Nihon Seibutsu Zairyo Centre, Japan. An anticoagulated blood was centrifuged at 2500 rpm for 5 min to obtain platelet-rich plasma (PRP). To perform the platelet adhesion examination, the Zein-SWCNT fibrous scaffold was placed onto a piece of flat glass. Then, a sample of 20 *μ*L of PRP was carefully dropped on the scaffold center. After incubation for 30 min at room temperature (about 37°C), the scaffold was carefully rinsed several times in phosphate buffer solution (pH-7.4) to remove non-adhering platelets. Adherent platelets on the scaffolds were preserved with 2.5% glutaraldehyde/PBS solution for 30 min, followed by dehydration procedure using a series of ethanol-water mixtures (0, 30, 50, 70, 90, 100 vol % of ethanol) for 30 min, respectively. For electron microscopy, samples were then air dried and coated with platinum and examined in a scanning electron microscope (JSM-7400F, JEOL, Japan).

## 3. Results and Discussion

### 3.1. Structural Characterization of Electrospun Zein-SWCNTs Nanofiber Membranes

**Figure 1:**
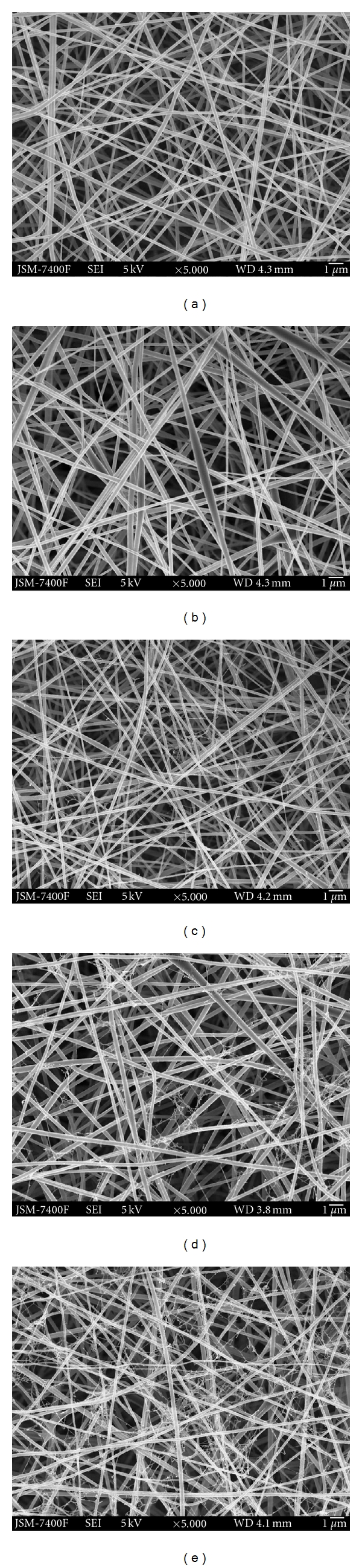
SEM image of electrospun (a) Zein nanofiber, (b) Zein-0.2 wt% SWCNTs, (c) Zein-0.5 wt% SWCNTs, (d) Zein-0.8 wt% SWCNTs, and (e) Zein-1 wt% SWCNTs nanocomposite scaffolds (scale Bar: 1 *μ*).

SWCNT-Zein solutions with varying SWCNT concentrations were prepared under sonication without phase separation and maintained homogenous solution. Due to the methods of dispersion of the SWCNTs by sonication and mechanical stirring, the SWCNT clusters break down. The addition of Zein forms a coating around each individual CNT and keeps them from aggregating or binding together. Solutions with 0 wt%, 0.2 wt%, 0.5 wt%, 0.8 wt%, and 1 wt% SWCNT content are spun according to the spinning conditions. Continuous fibers were obtained using a Zein concentration of 24 wt% and electrospinning at a distance of 10 cm under electric field strength of 2 kVcm^−1^. As can be seen from Figure 1(a), the absence of beads and the uniform fibers with smooth surface morphology was obtained for all experimental materials (Figures 1(b)–1(e)). Among them, the electrospun pure zein nanofibers Figure 1(a) had an average fiber diameter of 230 nm and diameter distribution for all loadings of SWCNT in the zein fibers (Figures 1(b)–1(e)) are all below the 300 nm range. However, at low concentration of nanotubes, relatively good dispersion was achieved, but as the concentration of nanotubes increased from 0.5 wt% to 0.8 wt% and 1 wt% SWCNTs promoted the creation of web-like structures in comparison with the zein nanofibers without SWCNTs. Random nanofiber structures and web-like structures can be obtained (Figures 1(d) and 1(e)), whereas the web-like structures consisted of regularly distributed very fine nanofibers with diameters of just about 20–40 nm, which were strongly embedded in the zein nanofibers. The web formation could be a result of the inclusion of carbon nanotubes into the fibers [41]. During electrospinning the SWCNTs are expelled from the polymer jet under extremely high force and velocity, which causes opening of the cluster to form the web-like structure. The Zein-SWCNT electrospun web like structure is very similar to the structure previously reported with using Bombyx mori silk nanofibers containing SWCNTs [42]. We do believe that these nanowebs were created because of strong secondary electric fields occurring between individual SWCNTs or their agglomerated form during the electrospinning process.

**Figure 2:**
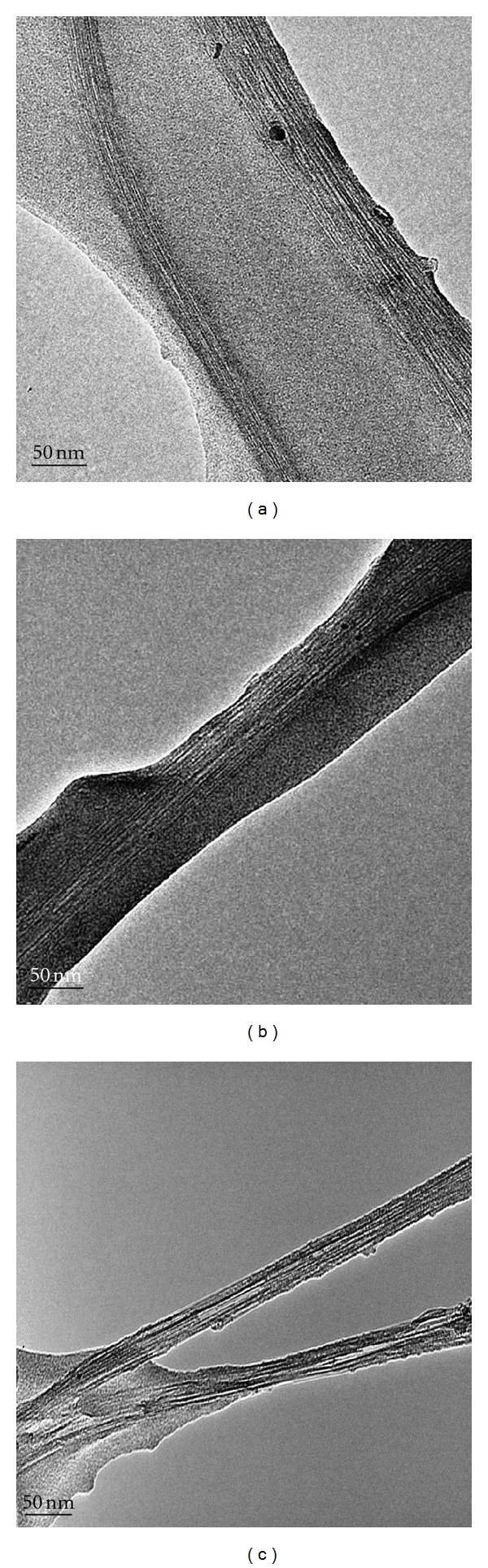
TEM micrographs of selected zein-SWNT composite nanofibers (Scale Bar: 50 nm).

TEM analysis of the Zein-SWCNT nanofibers prepared by the electrospinning process is provided in Figure 2 with the aim of analyzing the creation of the nanoweb fiber. The high resolution TEM micrograph shows the close inspection on electrospun composite fibers which indicates that the SWCNTs are embedded in the nanofibers without any sign of agglomerates. In many regions of the electrospun nanofibers; the embedded nanotubes appeared to be well oriented along the fiber axis. Such alignment is obviously associated with the extreme high longitudinal strain rate of jet during electrospinning process, which may cause disentanglement or pulling out of curved nanotubes under high shear force. SWCNTs were present inside the Zein nanofibers as individual tubes well aligned with nanofibers axes. Similar orientation of CNTs in the electrospun nanofibers has also been observed on other composite systems and it confirms that the original dispersion contains individual CNTs rather than aggregates or bulk [43–45].

### 3.2. Thermal Analysis for Nanocomposite Scaffold

**Figure 3:**
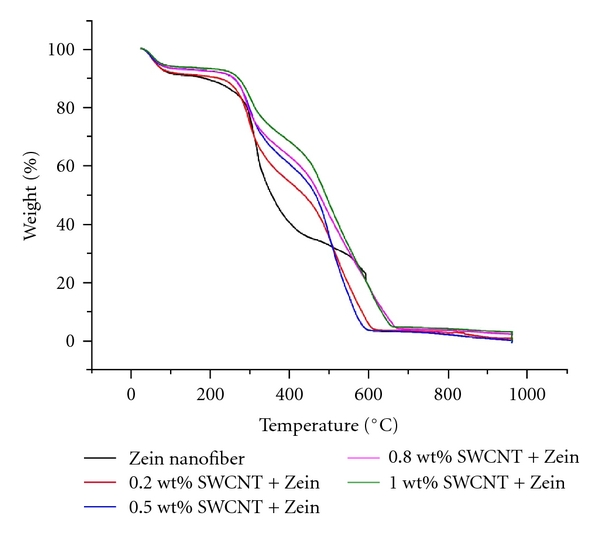
TGA thermograms of the zein nanofiber, zein-SWCNT nanocomposite scaffolds.

Thermogravimetric curves of electrospun Zein nanofiber and nanocomposite (Zein-SWCNT; 0.2–1 wt%) are shown in Figure 3. Thermal analysis shows that the improvement in thermal stability in case of Zein-SWCNTs nanocomposite than that of pure Zein nanofiber. The initial weight loss observed for all samples at 80°C is due to loss of moisture. The second weight loss occurs in the range of 230 to 380°C. This can be attributed to the breakdown of the amino acid residues, as well as cleavage of the peptide bonds. The initial degradation temperature is at 230°C and full degradation occurs at 455°C. From their degradation curves, it seems like the degradation of the SWCNT composite fibers occurs in two distinct phases. The electrospun Zein fibers on the other hand, have an initial degradation temperature of 280°C and final degradation temperature of 370°C at which the rate of mass loss is greatest. The 1 wt% SWCNT-Zein composite fiber has the highest thermal stability, with onset degradation at 250°C and full degradation occurring at 470°C.

### 3.3. Evaluation of Mechanical Properties of Nanocomposite Scaffold

Studying their mechanical properties can generate an idea about the structure of the nanocomposites. Du et al. [46] reported that the tensile strength, fracture toughness, and hardness tests showed improved properties up to 7 wt% of CNTs. When the CNT content exceeds 7 wt% the mechanical properties of the composites decreases. The composites become very brittle when CNTs get to 10 wt%. The tensile strength of PMMA by the addition of 3 wt% functionalized CNTs increased by 21%, which was low compared to a 90% increase in the modulus of PMMA by the addition of 2 wt% nonfunctionalized SWCNTs.

**Figure 4:**
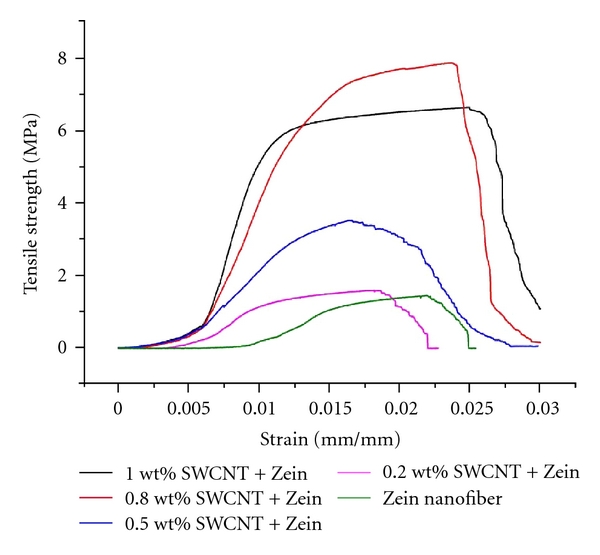
Typical tensile stress-strain curves of Zein nanofiber and Zein-SWCNTs nanocomposite Scaffolds.

Figure 4 illustrates the typical stress strain curves of Zein and Zein-SWCNTs composite nanofibrous scaffold. Pure Zein nanofiber shows a tensile strength of 1.4 MPa, while the tensile strength of Zein-SWCNTs nanofiber increased with increasing SWCNTs content (0.2; 0.5 and 0.8 wt%) to 3.2 MPa. Moreover, the Zein-0.8 wt% SWCNTs sample exhibited the maximum strength (about 7.7 MPa). Subsequently, further increasing the SWCNTs content (1 wt%) lead to the decrease of tensile strength (6.8 MPa) of Zein-SWCNTs nanofiber scaffold. It was observed that Zein nanofibrous scaffold with SWCNTs content higher than 1 wt% gives poor mechanical properties, which could be explained due to poor dispersions of the CNT within the fiber, result in forming defects of the scaffold. The above results indicate that the mechanical properties of Zein nanofiber can be improved by the addition of SWCNTs. The tensile tests have revealed that the scaffolds containing SWCNTs were able to withstand a higher stress than the corresponding unreinforced scaffolds. This can be explained taking into account the TEM images, which have clearly shown that the SWCNTs are aligned within and along the fiber orientation, thereby improving the mechanical properties. Nanomaterials have high surface energy and are easy to aggregate, which lead to the poor dispersion of nanomaterials in polymer matrix results in poor mechanical properties. Thus, good dispersion of SWCNTs in a polymer matrix the composite exhibited significant mechanical properties increased by incorporating the CNTs [47].

### 3.4. Water Uptake and Retention Abilities

**Figure 5:**
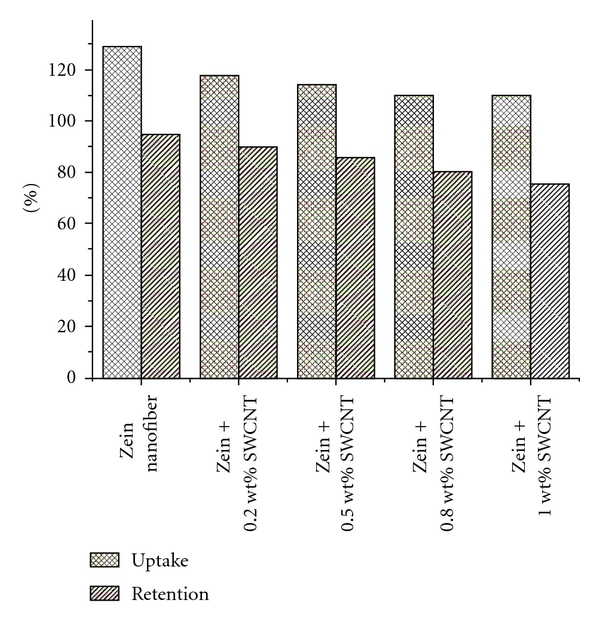
Water uptakes and water retention ability of zein scaffold, and Zein-SWCNT scaffolds after 24 hr. (*n* = 5).

The composite materials were expected to be used as the scaffolds; hence their water retention ability of the materials to keep water inside the matrix has an important role for the absorption of body fluid and for a transfer of cell nutrients and metabolites through the materials. Scaffolds showing higher degree of water uptake will have a larger surface area/volume ratio thus allowing the scaffold to have the maximum probability of cell growth in 3D scaffold [48]. The increase in water uptake also allows the scaffolds to avail nutrients from culture media more effectively [49]. Hence, controlled water uptake will be ideal for tissue engineering applications [50]. The results in Figure 5 give the water uptake ratios of five groups of scaffolds. The prepared 3D nanocomposite scaffolds based on Zein showed significant water uptake ability; the values were over 100%, and the highest value reached 128 ± 1.09% for Zein nanofiber. The composite nanofiber and native Zein nanofibers show the same general swelling trend. After 24 h of water absorption we have observed only 12% difference in the equilibrium water uptake capacity between native (128 ± 1.09%) and composite nanofiber (109 ± 0.45%) with 1 wt% of SWCNTs. These results demonstrate that the presence of SWCNTs has led to decrease on the water uptake capacity of the scaffold. 

The percent water retention values of the nanocomposite scaffolds were measured and showed in Figure 5. It was found that the use of SWCNT resulted in decrease of water retention. Water retention ability of Zein scaffold was higher compared to SWCNT-Zein scaffolds. We found that the values of water retention were still higher than 75% even if SWCNT was used, implying that the scaffolds could retain water whose weight was more than their own. Water retention meant that the scaffolds could absorb water to some extent and the tight aggregation of nanofibers might make the scaffold stable in sizes and shapes.

### 3.5. Hemolysis Analysis

In this research work, the hemocompatibility of Zein-SWCNTs nanofiber membranes were evaluated by hemolysis. The hemolysis percentage (HP) represents the extent of red blood cells hemolysis when they come in contact with sample. When the polymeric scaffold in contact with blood it must not induce thrombosis, thromboembolisms, antigenic responses, destruction of blood constituents, plasma proteins, and so forth [51]. Thus, biocompatibility, especially blood compatibility, is the most important property with regard to biomedical materials. Hence we had conducted the blood compatibility test for our nanocomposite scaffolds too. Results obtained for hemolysis of rabbit blood with nanocomposite scaffolds were shown in Table 1. Hemolysis occurs when red blood cells come into contact with water. The positive reference (100% lysis) was blood/water mixture and the negative reference (0% lysis) was a blood/saline mixture. The OD values of positive and negative were 0.9 and 0.02, respectively. Each absorbance data point was obtained by measuring three samples and also the deviations of the three tests were determined. However, Hemolysis was less than 4% for all tested Zein-SWCNTs scaffolds. This results well within the permissible limit set by Autian [52], who reported that a value of up to 5% hemolysis is permissible for biomaterials. The maximum HP value (4.0 ± 0.29%) was obtained for 1 wt% Zein-SWCNTs. When the SWCNTs content of the Zein was altered, no significant difference was observed in the hemolysis assay, indicating good character of antihemolysis among all the Zein-SWCNTs nanofiber Scaffolds (Table 1). It may be conclude from the hemolysis percentage that the Zein-SWCNT composite scaffold may be suitable as biomaterials for specific implant clinical application purpose.

### 3.6. Platelet Adhesion Examination

**Figure 6:**
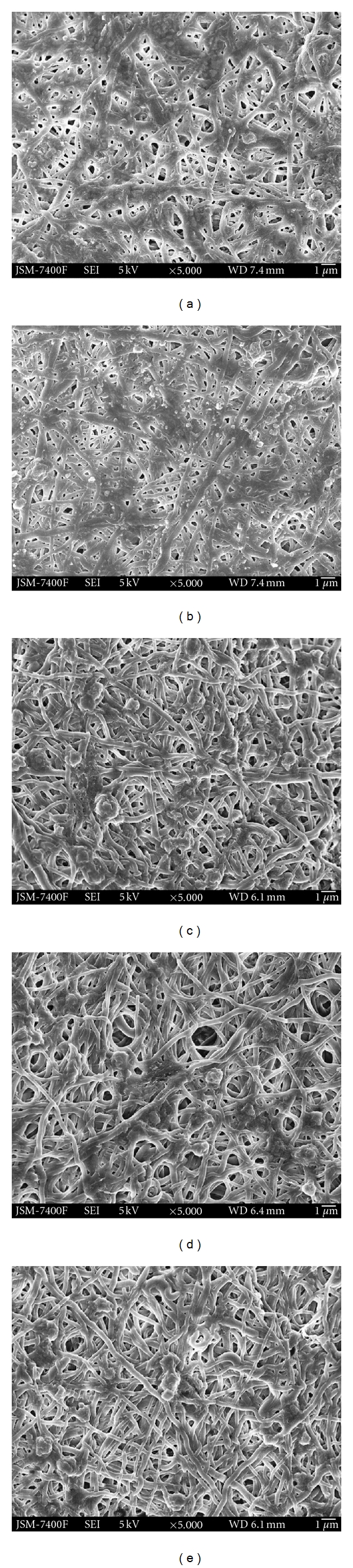
Effects of the composite nanofibers on the adhesion of blood platelets. (a) Zein nanofiber, (b) Zein-0.2 wt% SWCNTs, (c) Zein-0.5 wt% SWCNTs, (d) Zein-0.8 wt% SWCNTs, and (e) Zein-1 wt% SWCNTs nanocomposite Scaffolds (scale Bar: 1 *μ*).

To determine the hemocompatibility of the Zein-SWCNT composite nanofiber scaffolds, as is well known, platelet adhesion has generally been applied to evaluate the hemocompatibility of materials. When a foreign material comes into contact with blood, the initial blood response is the adsorption of blood proteins, followed by platelet adhesion and the activation of coagulation pathways, leading to thrombus formation [53]. The morphologies of activated platelets can be divided into 5 categories including dendritic, dendritic spread, spread, fully spread, and nonviable [54]. These criteria were used to assess the activation state of the platelets that adhered to the surface of Zein-SWCNT in the current study.  Figure 6 shows the electron micrographs of platelet adhesion on Zein and Zein-SWCNT composite nanofiber scaffolds. As can be seen from the photomicrographs, the both platelet adhesion and activation are observed on the Zein nanofiber (Figure 6(a)). The promotion of activation and adhesion of platelets was obviously due to the presence of nanofibers. 

We have observed a trend in the direction of decrease in platelet adhesion and absence of activation while increasing the SWCNTs and found very low in 1 wt% SWCNT-Zein composite (Figure 6(e)), which might be due to a lower water adsorption. However, on Zein nanofiber scaffold with a higher SWCNT content, some of the platelets have extended, but many retain a discoid shape which is similar to the original shape of the platelet and indicates an unactivated state. Changes in surface chemistry or roughness of sample surface will have an effect on the adsorption characteristic. Surfaces with negatively charged groups were reported to be antithrombogenic, whereas positively charged surfaces are thrombogenic [55]. We hypothesize that the decrease in the platelet adhesion on the Zein-SWCNT composite is influenced by the surface chemistry as well the surface morphology, where hydrophilic repulsion occurs and prevented the direct contact between the platelets with the Zein-SWCNT composite surface. These results may suggest that Zein-SWCNT nanofibrous composite containing 22 wt% zein and 0.8 wt% SWCNT could be a potential candidate for anti-thrombogenicity, which would be particularly useful for artificial blood prostheses.

## 4. Conclusion

We have developed nanofibrous nanocomposite scaffolds which were prepared by electrospinning of Zein and Zein-SWCNTs solutions. This method under sonication effectively dispersed SWCNTs in a continuous polymer scaffold. Microscopy images show good dispersion of SWCNTs and the composite exhibited significant mechanical properties as well as thermal stability of electrospun Zein-SWCNT nanocomposites. We have demonstrated that when the SWCNTs content of the Zein was altered, no significant difference was observed in the hemolysis assay, while it did alter the response in platelet adhesion with the absence of platelet activation on Zein-SWCNT composite. This suggested that platelet activation could be efficiently suppressed on this nanostructured composite with its excellent antiadhesion and subsequently low platelet activation. The developed Zein-SWCNT composite with homogeneous microstructure and improved mechanical properties can be used as a potential biomaterial for implant applications.

## Figures and Tables

**Table 1:** Hemolysis percentage of electrospun zein-SWCNTs electrospun nanocomposite scaffolds.

Sample	Optical density at 545 nm	Hemolysis (%)
Water (positive control)	0.912	—
Saline (Negative control)	0.002	—
Zein	0.025	2.5 ± 0.30
Zein + 0.2 wt% SWCNT	0.028	2.8 ± 0.30
Zein + 0.5 wt% SWCNT	0.030	3.0 ± 0.29
Zein + 0.8 wt% SWCNT	0.033	3.40 ± 0.25
Zein + 1.0 wt% SWCNT	0.039	4.0 ± 0.29
